# Chromosomal variability in Brazilian species of *Anthurium* Schott (Araceae): Heterochromatin, polyploidy, and B chromosomes

**DOI:** 10.1590/1678-4685-GMB-2018-0080

**Published:** 2019-11-14

**Authors:** Sarah do Nascimento, Marcus Alberto Nadruz Coelho, Joel M. P. Cordeiro, Leonardo P. Felix

**Affiliations:** 1 Laboratório de Citogenética Vegetal, Departamento de Ciências Biológicas, Centro de Ciências Agrárias, Universidade Federal da Paraíba, Areia, PB, Brazil.; 2 Instituto de Pesquisas, Jardim Botânico do Rio de Janeiro, Ministério do Meio Ambiente, Rio de Janeiro, RJ, Brazil.

**Keywords:** B chromosomes, CMA/DAPI, cytotaxonomy, Neotropical Araceae

## Abstract

The genus *Anthurium* has a Neotropical distribution, with karyotype predominance of *x* = 15, although some species show disploidy or polyploid variations. The karyotypes of seven species and different populations of *Anthurium* were analyzed using fluorochrome CMA and DAPI staining. The karyotypes were composed of meta- and submetacentric chromosomes, with numbers varying from 2*n* = 30 to 2*n* = 60. Supernumerary euchromatic chromosomes were observed in *A. affine*, and supernumerary heterochromatic chromosomes were observed in *A. gladiifolium* and *A. petrophilum*. Polyploidy was recurrent in the *Anthurium* species analyzed, with records of 2*n* = 30 and 60 in different *A. pentaphyllum* populations*.* Fluorochrome staining revealed different CMA^+^ banding distributions between diploid and polyploid cytotypes of *A. pentaphyllum*, suggesting structural alteration events. *Anthurium petrophilum*, on the other hand, showed a more consistent banding profile, with 10 to 12 proximal CMA bands in the three populations analyzed. DAPI^+^/CMA^0^ regions occurred exclusively in populations of *A. gracile* and *A. pentaphyllum*. The heterochromatic fraction in *Anthurium* was found to be quantitatively variable among species and populations, which may be related with adaptive aspects, different environmental conditions, or phylogenetic position.

## Introduction

The genus *Anthurium* is a strictly Neotropical monophyletic group that occurs from Mexico to Argentina, and is included within the subfamily Pothoideae, tribe Potheae (Croat, 1986; Coelho *et al.*, 2009; Cusimano *et al.*, 2011; Carlsen and Croat, 2013; Govaerts *et al.*, 2016). This group comprises approximately 950 species (Boyce and Croat, 2011 onwards), 134 of which are known in Brazil (Coelho *et al.*, 2018). The genus is taxonomically complex and subdivided into 18 sections (Croat and Sheffer, 1983) showing wide intra- and interspecific morphological diversity (Coelho and Mayo, 2007). The plants can have a terrestrial habit in the case of forest species, or be rupiculous, epiphytic, or hemiepiphytic vines, but they are rarely found in aquatic environments (Coelho *et al.*, 2009; Gonçalves and Jardim, 2009); there are numerous helophytic species that can be found growing on exposed rock surfaces (Gonçalves, 2005; Haigh *et al.*, 2011). The genus is monophyletic, with 18 clades that are easily distinguishable morphologically or geographically, and show low divergence in their *trnG* intron, *trnH-psbA* and *trnC-ycf6* sequences, and in the *CHS* intron regions of their DNA, suggesting a rapid radiation of the group (Carlsen and Croat, 2013).

Chromosomal records have been published for approximately 150 *Anthurium* species, with a predominance of 2*n* = 30 (88% of the species), but numbers vary from 2*n* = 24 to 2*n* = 124 (Sheffer and Croat, 1983; Petersen, 1989; Rice *et al.*, 2015). The genus is notorious for the occurrence of intraspecific polyploidy, such as *A. bellum* Schott with 2*n* = 30, 90 (Sheffer and Croat, 1983; Cotias-de-Oliveira *et al.*, 1999), *A. pentaphyllum* G.Don with 2*n* = 30, 60 (Cotias-de-Oliveira *et al.*, 1999), and *A. digitatum* (Jacq.) G.Don with 2*n* = 30, 60 (Rice *et al.*, 2015). Six species from southeastern and southern Brazil were analyzed and intraspecific polyploidy was observed in three of them, with the predominance of diploid cytotype, as in *A. urvilleanum* Schott and *A. harrisii* G.Don, or the predominance of tetraploid cytotype, as in *A. intermedium* Kunth (Viégas *et al.*, 2006). B chromosomes also occur frequently and have been observed in both diploid and tetraploid samples. A population of *A. urvilleanum*, close to Paratí, Rio de Janeiro State, Brazil (M. Nadruz, 1543), showed 2*n* = 30 + 0-2Bs, while another population within the same municipality (M. Nadruz, 1394) showed 2*n* = 60 + 0-2Bs (Viégas *et al.*, 2006). In spite of the frequent presence of B chromosomes in *Anthurium*, they are not easily distinguishable based on size and shape, or methodology employed.

The most complete study of B chromosomes in *Anthurium* was undertaken by Marutani and Kamemoto (1983), and included examining both somatic and meiotic cells in *A. warocqueanum* Moore. These authors observed that the numbers of B chromosomes in somatic cells in the species was constant (2*n* = 30 + 3B), although there were different associations during metaphase I of meiosis (one trivalent, one bivalent and one univalent, or three univalents), resulting in variable numbers of B chromosomes in selfed offspring (ranging from 0 to 6) and indicating their transmission from both male and female gametes. The diversity of B chromosomes in *Anthurium* was noted by Marutani *et al.* (1993), who reported them in *A. ochranthum* K.Koch, *A. cerrocampanense* Croat, and *A. garagaranum* Standl*.*, as well as in at least six interspecific hybrids resulting from crosses between *A. kamemotoanum* Croat *A. ochranthum*, *A. lindenianum* K.Koch & Augustin *A. cerrocampanense*, *A. garagaranum*  *A. lindenianum*, *A. cerrocampanense*  *A. garagaranum*, *A. formosum* Schott *A. cerrocampanense*, and *A. subsignatum* Schott *A. garagaranum*.

B chromosomes are commonly heterochromatic, although they appear euchromatic in some species (Camacho *et al.*, 2000; Banaei-Moghaddam *et al.*, 2014). No differential staining of *Anthurium* chromosomes has yet been undertaken, and the chromatin compositions of B chromosomes among its different species have not been examined. We therefore analyzed chromosome number variability and CMA/DAPI banding distributions in seven Brazilian species of *Anthurium* to identify interspecific variations and supernumerary chromosomes in different populations and cytotypes. The main objective of this work was to identify karyotype variability in Brazilian species of *Anthurium* to determine the importance of that variability to chromosome evolution in the genus.

## Materials and Methods

### Collections and botanical documentation

Seven species of *Anthurium* harvested in various regions of Brazil were investigated, including individuals from three different populations. Intraspecific variations were investigated in four of the seven species. Information concerning all of the samples and their respective collection localities, populations, and collectors are summarized in Table 1. Specimens were maintained alive in the experimental gardens of the Plant Cytogenetic Laboratory of the Department of Biological Sciences of the Agrarian Sciences Center at the Federal University of Paraíba (UFPB), Brazil. Exsiccates were deposited in the Prof. Jayme Coelho de Moraes Herbarium (EAN).

**Table 1:** The *Anthurium* species analyzed, citing their origins, voucher numbers, chromosome numbers, CMA/DAPI bands, and figures.

Genus/species	Voucher	Origin (city/state)	2*n*	CMA bands	Figures
*Anthurium affine* Schott	LPFelix 14635	Queimadas, PB	30 + 1B	2p	1A
	EMAlmeida 453	Águas Belas, PE	30	1p	1B
			30 + 2B	2p	1C
	EMAlmeida 476	Andaraí, BA	30	2p	1D
			30 + 3B	2p	1E
*A. gladiifolium* Schott	JPCastro 40	Jacobina, BA	30 + 3B	12p	1F
*A. gracile* Lindl.	JPCastro 61	Senhor do Bonfim, BA	30	10p	1G
	LPFelix 13662	Mamanguape, PB	40	2p	1H
	LPFelix 14865	Peruíbe, SP	40	2p	1I
*A. jilekii* Schott*	LPFelix 13761	Taquaritinga do Norte, PE	30	2p	2A
*A. pentaphyllum* G.Don	LPFelix 15074	Meruoca, CE	30	13p	2B
	LPFelix 13663	Mamanguape, PB	60	5p	2C
	LPFelix 14871	Itabaiana, SE	60	3p	2D
*A. petrophilum* K.Krause*	LPFelix 12614	Buíque, PE	30	10p	2E
	EMedeiros-Neto 22	Brejo da Madre de Deus, PE	30	12p	2F
	SNascimento 150	São João do Tigre, PB	30 + 1B	12p	2G
*Anthurium* sp.	LPFelix 15273	São Roque de Minas, MG	30	2p	2H

### Chromosomal analyses

Root tips were pretreated with 0.2% colchicine for 24 h at 10 C, fixed in 3:1 ethanol – acetic acid (v:v) for 2 h at room temperature, and subsequently stored at -20 C until analyzed. The material was then washed in distilled water and digested in an enzymatic solution containing 2% cellulase (Onozuka) and 20% pectinase (Sigma) (w/v) for 1 h at 37 °C. Slides were prepared using the squashing method in a drop of 45% acetic acid. Coverslips were subsequently removed in liquid nitrogen and samples were then air dried and kept for three days at room temperature (Guerra and Souza, 2002).

Fluorochrome staining followed the protocol described by Carvalho *et al.* (2005). Samples were stained with 10 μL chromomycin A3 (CMA) (0.1 mg/mL) and stored for 1 h in the dark, before staining with 10 μL de DAPI (2 μg/mL), were again stored in the dark for 30 min before mounting with glycerol/Mcllvaine. The slides were aged for three days in the dark to stabilize the fluorochromes. Metaphases were photographed using a AxioCam MRm epifluorescence microscope (Zeiss) equipped with a video camera, utilizing Axiovision 4.8 software (Zeiss). Images were processed using Adobe Photoshop CS3 Software (Adobe Systems). Chromosome measurements were made using Image Tool 3.0 software (Brent *et al.*, 2008). Chromosome morphology was determined using the centromeric index, following Guerra (1986a).

## Results

**Figure 1:**
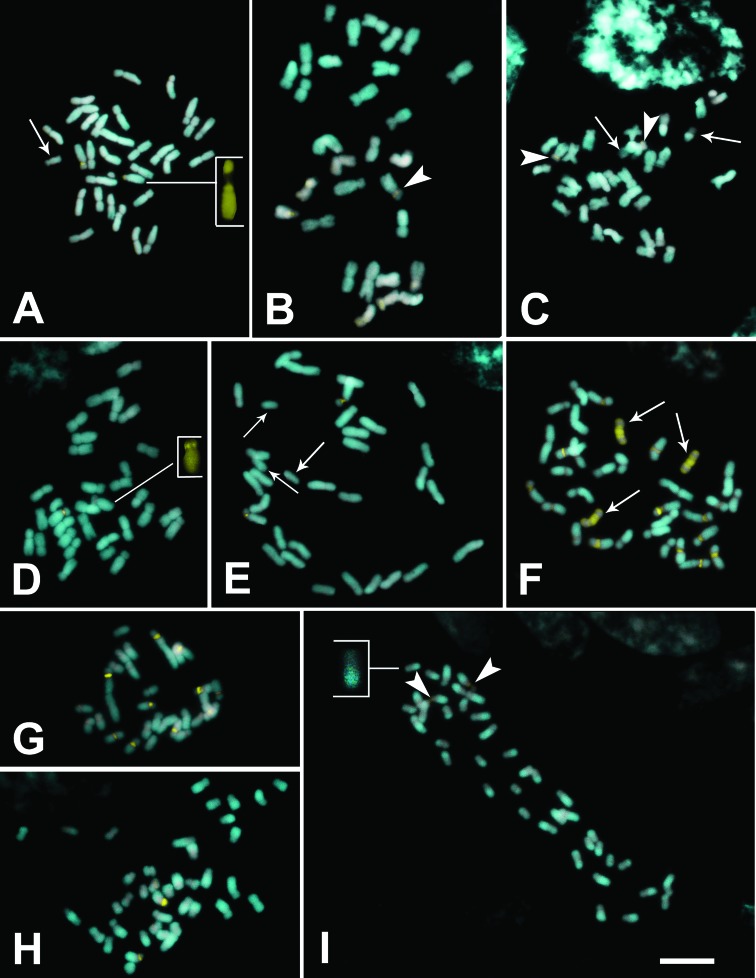
Metaphases of *Anthurium* species under CMA/DAPI staining. (A-E) *Anthurium affine*. (A) Queimadas-PB population with 2*n* = 30+1B; (B-C) Águas Belas-PE population, with 2*n* = 30 (B) and 2*n* = 30+2Bs (C); (D-E) Andaraí-BA population with 2*n* = 30 (D) and 2*n* = 30+3Bs (E); (F) *A. gladiifolium* (2*n* = 30+3Bs); (G-I) *A. gracile*. Senhor do Bonfim-BA population with 2*n* = 30 (G), Mamanguape-PB (H) and Peruíbe-SP population (I), both with 2*n* = 40. Arrows in A, C, E and F show B chromosomes; arrow heads in B, C and I show minor CMA bands; inserts in A and D highlight chromosomes with CMA bands; inserts in I show chromosomes with DAPI^+^/ CMA^0^ blocks. Bar in I is equivalent to 10 μm.

**Figure 2:**
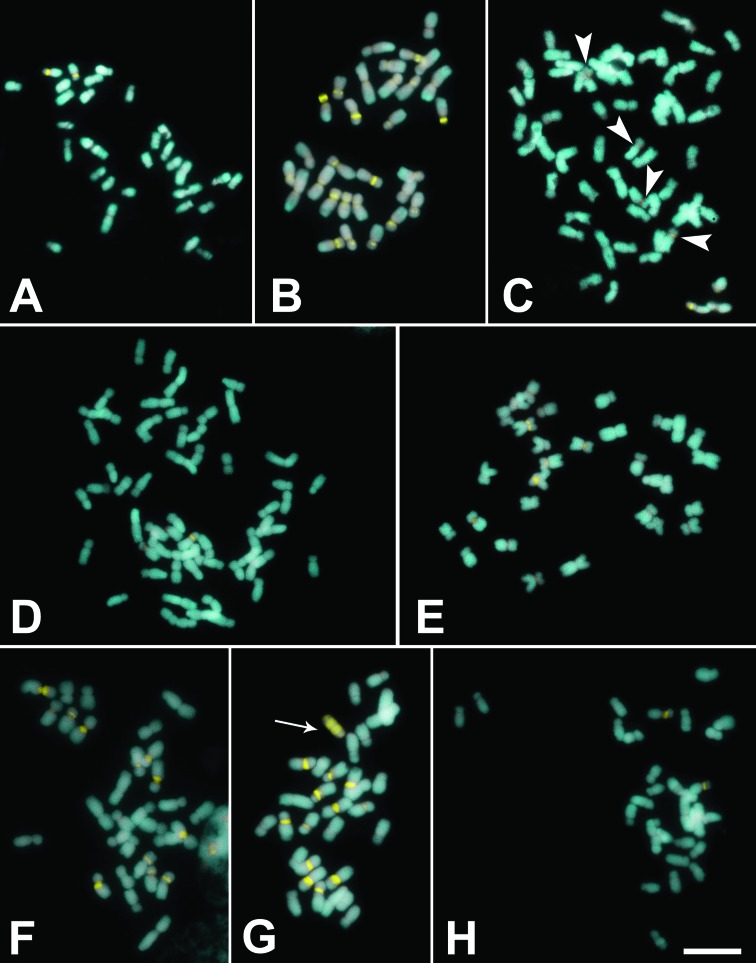
Metaphases of *Anthurium* species under CMA/DAPI staining. (A) *Anthurium jilekii* with 2*n* = 30; (B-D) *A. pentaphyllum*, Meruoca-CE population with 2*n* = 30 (B), Mamanguape-PB (C) and Itabaiana-SE populations (D), both with 2*n* = 60; (E-G) *A. petrophilum*, Buíque-PE population (E) and Brejo da Madre de Deus-PE population (F), with 2*n* = 30 and São João do Tigre-PE population (G) with 2*n* = 30+1B; H. *Anthurium* sp. (2*n* = 30). Arrows in G show B chromosomes; arrow heads in C show chromosomes with CMA bands. Bar in H is equivalent to 10 μm.

Chromosome numbers and heterochromatin characteristics are summarized in Table 1. All species exhibited symmetrical karyotypes, with chromosomes varying from submetacentric to metacentric (Figures 1 and 2). Chromosome numbers varied from 2*n* = 30 to 2*n* = 60, with most species showing 2*n* = 30; 2*n* = 40 was observed in two populations of *A. gracile* (Figure 1H, I) and 2*n* = 60 in two populations of *A. pentaphyllum* (Figures 2C-D). Euchromatic B chromosomes were observed in a population of *A. affine* Schott from Queimadas, Paraíba State (Figure 1A), and in populations from Águas Belas, Pernambuco State (Figure 1C) and Andaraí, Bahia State (Figure 1E). *Anthurium gladiifolium* Schott, on the other hand, showed three heterochromatic B chromosomes (Figure 1F), while the population of *A. petrophilum* K.Krause from São João do Tigre, Paraíba, showed a single heterochromatic B chromosome (Figure 2G). The species of *Anthurium* with B chromosomes analyzed here, their respective populations, and the frequency of B chromosomes in mitotic cells are presented in Table 2. None of the other species exhibited supernumerary chromosomes.

**Table 2:** *Anthurium* species with B chromosomes and their frequencies of occurrence in mitotic cells.

Species	Origin (city/state)	Total number of analyzed cells	2*n*	Frequency absolute (relative)
*Anthurium affine*	Queimadas, PB	13	30 + 1B	13 (100%)
	Águas Belas, PE	15	30	9 (60%)
	30 + 2B	6 (40%)		
	Andaraí, BA	30	30	11 (36.7%)
	30 + 3B	19 (63.3%)		
*A. gladiifolium*	Jacobina, BA	31	30 + 3B	31 (100%)
*A. petrophillum*	São João do Tigre, PB	35	30 + 1B	35 (100%)

Staining with fluorochromes revealed from one to two proximal CMA^+^/DAPI^–^ bands on the short arm of *A. affine* (Figure 1A-E), in two populations of *A. gracile* Lindl. with 2*n* = 40 (Figure 1H-I), in *A. jilekii* Schott (Figure 2A), and in *Anthurium* sp. (Figure 2H). The population of *A. gracile* from Senhor do Bonfim, Bahia (2*n* = 30) showed up to 10 conspicuous proximal CMA bands (Figure 1G), while *A. gladiifolium* showed up to 12 proximal bands (Figure 1F), and up to 13 CMA bands were seen in a diploid population of *A. pentaphyllum* (Figure 2B). The tetraploid populations of *A. pentaphyllum* examined, however, exhibited five proximal CMA bands in a population from Mamanguape, Paraíba (Figure 2C) and three bands in a population from Itabaiana, Sergipe (Figure 2D). *Anthurium petrophilum*, on the other hand, demonstrated a more consistent banding profile, with 10 to 12 proximal CMA bands in the three populations analyzed. DAPI^+^/CMA^–^ bands were not clearly observed, except in the *A. gracile* population from Peruíbe (Figure 1I) and in *A. pentaphyllum* from Meruoca (Figure 2B), where the terminal regions of some chromosomes appeared stained with DAPI rather than with CMA, which were interpreted as DAPI^+^/CMA^0^ regions.

## Discussion

### Numerical chromosome variations

Of the seven species analyzed here, our results confirmed previous counts for *A. pentaphyllum* with 2*n* = 30 and 60 (Cotias-de-Oliveira *et al.*, 1999), *A. gracile* with 2*n* = 30 and 40 (Sheffer and Kamemoto, 1976; Guerra, 1986b), and *A. gladiifolium* and *A. affine* with 2*n* = 30 (Sheffer and Kamemoto, 1976; Carvalheira *et al.*, 1991; Cotias-de-Oliveira *et al.*, 1999). The counts for *A. jilekii* and *A. petrophilum*, both with 2*n* = 30, are new.

The chromosome number 2*n* = 30 is the most frequent in the genus *Anthurium*, although other chromosome numbers, such as 2*n* = 26, 28, 32, 36, and 40, also occur (Sheffer and Kamemoto, 1976; Sheffer and Croat, 1983; Viégas *et al.*, 2006). Those variations may represent cases of ascending or descending disploidy or different euploidy series of *n* = 15. Similarly, reports of polyploidy generally follow two distinct models (2*n* = 30-60-90 and 2*n* = 28-56) (Sheffer and Croat, 1983; Viégas *et al.*, 2006). Among the polyploid species analyzed, *A. pentaphyllum* follows the 30-60-90 model, the most common in the genus (Sheffer and Croat, 1983). In the 2*n* = 30, 40 and 60 series reported for *A. gracile* (Sheffer and Croat, 1983; Guerra, 1986b; present work), however, 2*n* = 40 may have resulted from ascending or descending disploidy, although there are no intermediate chromosome numbers in the literature in support of those events.

Polyploidy and disploidy are among the most important karyotype phenomena associated with the evolution of plant groups (Stebbins, 1971; Soltis *et al.*, 2014). Species showing disploidy and polyploidy tend to be morphologically distinct from their parental diploids, and can present adaptations to different habitats and ecological niches (Madlung, 2013; Ramsey and Ramsey, 2014; Cordeiro *et al.*, 2018; Scholthof *et al.*, 2018). However, the occurrence of disploidy or polyploidy in *Anthurium* does not appear to have any apparent ecological correlation or link to their geographic distributions, although they may have considerable effects on speciation within the genus, especially in the species *A. scandens* (Aubl.) Engl. (2*n* = 24, 48, 84), *A. digitatum* (Jacq.) G.Don (2*n* = 26, 30, 36, 40, 60), and *A. bellum* Schott (2*n* = 28, 56, 90) (Sheffer and Kamemoto, 1976; Rice *et al.*, 2015).

The basic number *x* = 15 appears as the most probable for *Anthurium* based on the wide occurrence of 2*n* = 30 in the genus (Marchant, 1973). Sheffer and Kamemoto (1976) and Sheffer and Croat (1983) suggested *x* = 12 as the basic ancestral number due to records of 2*n* = 24 and 48 in species of the section *Tetraspermium* Schott. Molecular phylogenetic data nonetheless suggest that the section *Tetraspermium* occupies a derived position in the genus (Carlsen and Croat, 2013). *Anthurium flexile* Schott, with 2*n* = 60 (Sheffer and Kamemoto, 1976), and *A. clidemioides* Standl. with 2*n* = 30 (Petersen, 1989) have been considered the most basal species (Carlsen and Croat, 2013), in support of *x* = 15 as the basic number of *Anthurium*. However, the hypothesis of *x* = 12 cannot be discarded offhand, as species of the genus *Pothos* L. (a sister group to *Anthurium*) show 2*n* = 24 and 26 (Rice *et al.*, 2015), suggesting a relationship of those numbers to the karyotypic evolution of *Anthurium*.

### B chromosomes

Of the 153 species of the genus *Anthurium* with known chromosome numbers, B chromosomes have been identified in 20 (approximately 13%). Among the species found to have B chromosomes, there are records for *A. affine* (Cotias-de-Oliveira *et al.*, 1999) and the new occurrences in *A. gladiifolium* (30+3Bs) and *A. petrophilum* (30+1B). However, the occurrence of B chromosomes in the genus may be underestimated, whereas the numbers of B chromosomes may have been interpreted as intraspecific disploidy variation in chromosome numbers. For example, *A. obtusum* (Engl.) Grayum with 2*n* = 24, 30, *A. durandii* Engl. with 2*n* = 28, 30 (Sheffer and Croat, 1983), and *A. conspicuum* Sodiro with 2*n* = 28, 32 (Rice *et al.*, 2015) may reflect B chromosomes interpreted as A chromosomes.

The B chromosomes of *Anthurium*, besides varying in number, can also vary in their origin and chromatin composition. *Anthurium affine* is distinct from other species because its B chromosomes were euchromatic, while *A. gladiifolium* and *A. petrophilum* show B chromosomes composed principally of GC-rich heterochromatin. *Anthurium affine* is characterized by having only small quantities of GC-rich heterochromatin, which are observed only in the NORs of one or two chromosomes. *Anthurium gladiifolium* and *A. petrophilum*, on the other hand, show large CMA bands in the pericentromeric regions of five to six chromosome pairs. Although the origins of B chromosomes are not yet certain, one well-accepted hypotheses is their derivation from A chromosomes (Jones and Houben, 2003; Houben *et al.*, 2013). In that sense, it is reasonable to suppose that GC-rich heterochromatin regions of the A chromosomes of *A. gladiifolium* and *A. petrophilum* were incorporated into (and amplified in) their B chromosomes.

The occurrence of B chromosomes in *Anthurium*, as well as other groups of plants, seems to be independent phenomena (Camacho *et al.*, 2000; Levin *et al.*, 2005), without any clear effects above the species level. Phylogenetic analyses corroborate that hypothesis, as one can see in *Anthurium* species that have B chromosomes, but are placed in different clades (see the phylogenetic hypothesis proposed by Carlsen and Croat, 2013). As in *Anthurium*, the occurrences of B chromosomes in *Picea* A.Dietr. (Pinaceae) do not show clear phylogenetic relationships (Lockwood *et al.*, 2013). All of the species of *Calochortus* Pursh (Liliaceae) that have B chromosomes (D’Ambrosio *et al.*, 2017), on the other hand, are in the same clade (Subsection *Venusti*, Patterson and Givnish, 2003), suggesting that the occurrence of B chromosomes in different plants reflects different causes.

The presence of B chromosomes can produce phenotypic effects at the level of individuals, especially related to vigor, fertility and fecundity, increased germination vigor or speed, or the appearance of morphological traits (leaf striping in maize, for example) (Camacho *et al.*, 2000; Banaei-Moghaddam *et al.*, 2014; Houben *et al.*, 2014). Studies involving correlations of B chromosomes and ecological/adaptive aspects will be extremely important to the understanding of their evolutionary relationships in plants, making *Anthurium* an excellent genus for testing hypotheses.

### Heterochromatin in Anthurium

Heterochromatin distribution appears to be relatively variable among different species and populations of *Anthurium*. Heterochromatin is most frequently located in the subtelomeric and pericentromeric regions of plant chromosomes and in NORs (Lamb *et al.*, 2007). Heterochromatin associated with NORs in plants frequently appears as CMA^+^/DAPI^–^ bands (Guerra, 2000). Those sequences can be differentially amplified, forming characteristic patterns useful in differentiating between the karyotypes of closely related taxa, such as in *Citrus* L. (Carvalho *et al.*, 2005), *Acianthera* Scheidw. (Oliveira *et al.*, 2015), the Bignonieae tribe (Cordeiro *et al.*, 2017), *Spondias* L. (Almeida *et al.*, 2007), *Ameroglossum* Eb. Fisch., S. Vogel & A.V.Lopes (Almeida *et al.*, 2016), and *Vigna* Savi (Shamurailatpam *et al.*, 2014). Differential amplification of heterochromatin was observed in all of the species analyzed in the present work, especially in *A. gracile*, which exhibited from 2 to 10 CMA^+^ bands in different populations.

The phenomena responsible for variation in the heterochromatic portions of different plant species are not well known. The diverse CMA banding patterns observed in genera such as *Caesalpinia* L. *sensu latu* (Fabaceae) appear to be related to geographic distribution, ecological niches, and the phylogenetic relationships between the species (Van-Lume *et al.*, 2017). The heterochromatic fraction in *Anthurium* is quantitatively variable among species and populations, and may be related to adaptive aspects, reflecting environmental or phylogenetic factors in those taxa. Corroborating this hypothesis, the population of *A. gracile* from Senhor do Bonfim in the semiarid region of Bahia showed large numbers of CMA bands (10) when compared to populations from the humid coastal areas of Paraíba and São Paulo (each with only one pair of bands). Chromosome studies involving larger numbers of species and populations, in conjunction with evolutionary phylogenetic methodologies, could aid in understanding the karyotypic diversity observed in *Anthurium,* one of the most diversified groups of Neotropical monocotyledons.
